# Correlation between oxidative stress indices and CD4 counts in HIV infected patients

**DOI:** 10.1186/1471-2334-12-S1-P71

**Published:** 2012-05-04

**Authors:** Vamseedhar Annam, DR Suresh

**Affiliations:** 1 Department of Pathology, Melmaruvathur Adhiparasakthi Institute of Medical Sciences & Research, Melmaruvathur, Tamil Nadu, India; 2 Department of Biochemistry, ESI-PGIMSR, Bangalore, Karnataka, India

## Background

HIV infection induces wide array of immunologic alterations resulting in progressive development of AIDS. Of the mechanisms contributing to this progression, oxidative stress induced by the production of reactive oxygen species may play a critical role in the stimulation of HIV replication and development of immunodeficiency. This study was conducted to measure total antioxidant capacity and lipid peroxidation in HIV patients and correlate with their CD4 counts.

## Methods

The present cross-sectional study comprised of 4 groups. Group-1: Normal controls (n=100), Group-2: HIV patients with CD4 count > 500 cells/micro liter (n=100), Group-3: HIV patients with CD4 count between 200-499 cells/micro liter (n=100) and Group-4: HIV patients with CD4 count < 200 cells/micro liter (n=100). The CD4 counts were performed by Tricolor Flowcytometer. Evaluation of lipid peroxidation was performed by estimating serum malondialdehyde, while the marker for total antioxidant capacity was performed by FRAP assay. The oxidative stress index was measured. The statistical significance was determined by unpaired Student’s t-test and Pearson’s correlation coefficient at 5% level significance.

## Results

There was statistically significant negative correlation found between CD4 counts and MDA [p value <0.001] and significant positive correlation was found between CD4 counts and TAC in HIV-infected patients [p value <0.001].

## Conclusion

The oxidative stress markers were inversely proportional to CD4 counts in HIV patients reflecting the increased formation of ROS and lipid peroxidation with progressive immunodeficiency. The TAC levels were directly proportional to CD4 counts. Thus, antioxidant therapy can be useful to monitor and optimize the management of HIV-infected patients.

